# Structural Basis for the Site-Specific Incorporation of Lysine Derivatives into Proteins

**DOI:** 10.1371/journal.pone.0096198

**Published:** 2014-04-23

**Authors:** Veronika Flügel, Milan Vrabel, Sabine Schneider

**Affiliations:** 1 Department of Chemistry, TU Munich, Garching, Germany; 2 Department of Chemistry, Ludwig-Maximilians University, Munich, Germany; Berlin Institute of Technology, Germany

## Abstract

Posttranslational modifications (PTMs) of proteins determine their structure-function relationships, interaction partners, as well as their fate in the cell and are crucial for many cellular key processes. For instance chromatin structure and hence gene expression is epigenetically regulated by acetylation or methylation of lysine residues in histones, a phenomenon known as the ‘histone code’. Recently it was shown that these lysine residues can furthermore be malonylated, succinylated, butyrylated, propionylated and crotonylated, resulting in significant alteration of gene expression patterns. However the functional implications of these PTMs, which only differ marginally in their chemical structure, is not yet understood. Therefore generation of proteins containing these modified amino acids site specifically is an important tool. In the last decade methods for the translational incorporation of non-natural amino acids using orthogonal aminoacyl-tRNA synthetase (aaRS):tRNAaaCUA pairs were developed. A number of studies show that aaRS can be evolved to use non-natural amino acids and expand the genetic code. Nevertheless the wild type pyrrolysyl-tRNA synthetase (PylRS) from *Methanosarcina mazei* readily accepts a number of lysine derivatives as substrates. This enzyme can further be engineered by mutagenesis to utilize a range of non-natural amino acids. Here we present structural data on the wild type enzyme in complex with adenylated ε-*N*-alkynyl-, ε-*N*-butyryl-, ε-*N*-crotonyl- and ε-*N*-propionyl-lysine providing insights into the plasticity of the PylRS active site. This shows that given certain key features in the non-natural amino acid to be incorporated, directed evolution of this enzyme is not necessary for substrate tolerance.

## Introduction

**Figure 1:**
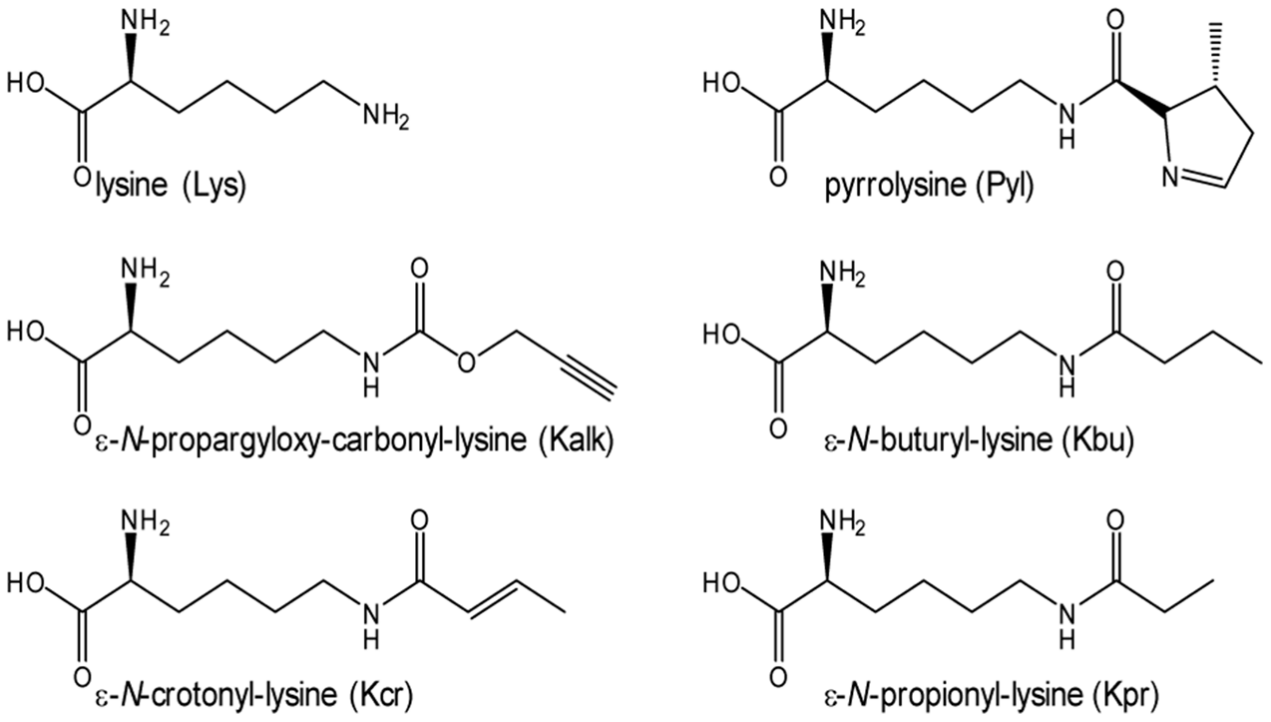
Lysine, pyrrolysine and analogs used in this study.

The regulation of many cellular key processes, such as gene expression, protein activity and stability as well as molecular recognition relies on the posttranslational modification (PTM) of proteins. Lysine is the main target for PTMs particularly in the context of chromatin structure, remodeling and thus epigenetic gene regulation, known as the ‘histone code’ [1], [2]. Histone acetyltransferases (HATs) and histone deacetylases (HDACs), which are enzymes responsible for lysine modifications, are regarded as important drug targets [3]. Recently it became clear that lysine residues in histones can not only be acetylated or methylated, but also malonylated, succinylated, butyrylated, propionylated and crotonylated [4], [5], [6], [7], [8], [9]. These modifications change the net charge of the residue from positive to negative or neutral as well as alter the hydrophobicity and flexibility of the modified protein. The interaction properties, landscape and partners of the protein are therefore altered, ultimately impacting on chromatin structure and gene expression [2]. Despite the fact that some of the lysine modifications like buturylation, crotonylation and propionylation are very similar in their chemical structure (Fig. 1), their effect on gene expression differ depending on the context. For instance, a direct link between activation of gene expression through crotonylation of Lys on histone H3 has been demonstrated [6], [10]. Additionally, various human diseases like cancer [11], disorders of the central nervous system [12], and autoimmune diseases [13] are associated with misregulation of histone PTMs. Lysine PTMs are not restricted to histones – for example, the tumor suppressor protein p53 is regulated by phosphorylation and ubiquitination at its C-terminal lysine residues [14], [15]. In order to be able to understand the epigenetic regulation of cellular key processes, it is essential to elucidate the distinct temporal and special patterns of PTMs. Thus, lysine modifying enzymes, interaction partners and their regulatory proteins need to be identified. It is therefore crucial to generate the target protein containing site-specific modifications. In the last decade methods to genetically encode amino acids beyond the canonical 20 amino acids have been developed. Here orthogonal aminoacyl-tRNA synthetase (aaRS):tRNAaaCUA pairs are used, such as the pyrrolysyl tRNA synthetase (PylRS) from *Methanosarcina mazei*, *Methanosarcina barkeri* and tyrosyl-tRNA synthetase (TyrRS) from *Methanococcus jannaschii*, which incorporate pyrrolysine (Pyl) and tyrosine. A number of studies show that aaRS can be further evolved to accept non-natural, functionalized or chemical modifiable amino acids. By introducing an amber stop codon in a given gene, the host's endogenous translational machinery can be employed to incorporate non-natural amino acids site-specifically, thus allowing the functionalization of the target protein [16], [17], [18], [19], [20], [21], [22], [23], . Structures of PylRS in complex with its natural substrate [25], [26], [27] as well as evolved mutants containing non-natural amino acids are already available [28], [29], [30]. Using an evolved PylRS (L274A C313A Y349F) from *M. barkeri* ε-*N*-crotonlyl-lysine was introduced into histones [17]. However the wild type PylRS from *M. mazei*, which shares 71% sequence identity (85% sequence similarity) with the homologue from *M. barkeri*, readily utilizes ε-*N*-butyryl-(Kbu), Kcr and ε-*N*-propionyl-lysine (Kpr) as substrates (Fig. 1) and can therefore be used directly to incorporate these different PTMs into histones [31]. In addition, ε-*N*-propargyloxy-carbonyl-lysine (Kalk) is also accepted, which allows functionalization after heterologous expression using Cu(I)-catalyzed azide-alkyne cycloaddition reaction [32], [33], [34], [35], [36], [37]. Here we present the X-ray crystal structures of the PylRS from *M. mazei* in complex with adenylated Kalk, Kbu, Kcr and Kpr (Fig. 1). The data show that the amino acids are held in the PylRS active site such that variations at the ε-*N* position are allowed and key features can be deduced. The factors limiting the derivatives that can be accommodated by the wild type PylRS are length, flexibility and charge properties: The binding pocket is predominantly lined by hydrophobic residues and analogues which are extended compared to Pyl, but flexible can ‘curl up’ in order to fit in the active site. Taken together this provides insights into the plasticity of the active site of this enzyme and shows that non-natural amino acids with certain key features can be readily incorporated. Thus PylRS evolution to utilize such non-natural amino acids for the incorporation in a given target protein is not strictly necessary.

## Results and Discussion

### Structures of lysine derivatives bound by the catalytic domain of PylRS

**Figure 2:**
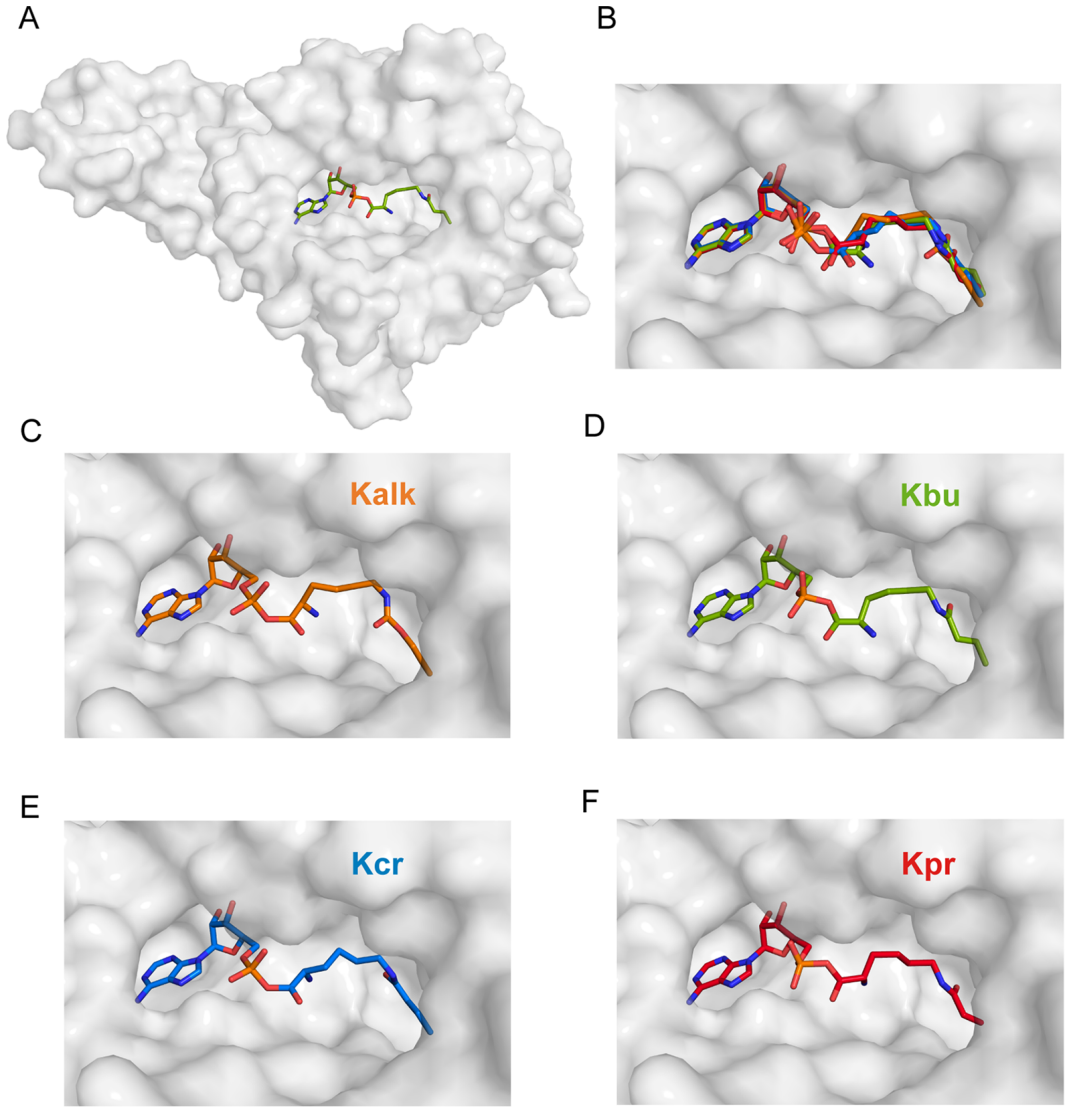
Cartoon representation of the overall structure of the catalytic domain of PylRS. (A) Type-II tRNA-synthetase folding topology of the tRNA synthetase domain from PylRS, shown as cartoon representation, overlaid with its semi-transparent surface. The adenylated Kbu is highlighted as green stick model in the active site. (B-F) Zoom in the active site: PylRS in complex with Kalk (orange), Kbu (green), Kcr (blue) and Kpr (red), drawn as stick models. (r. m. s. d ∼0.35 Å). All four non-natural amino acids bind in the same position.

**Figure 3:**
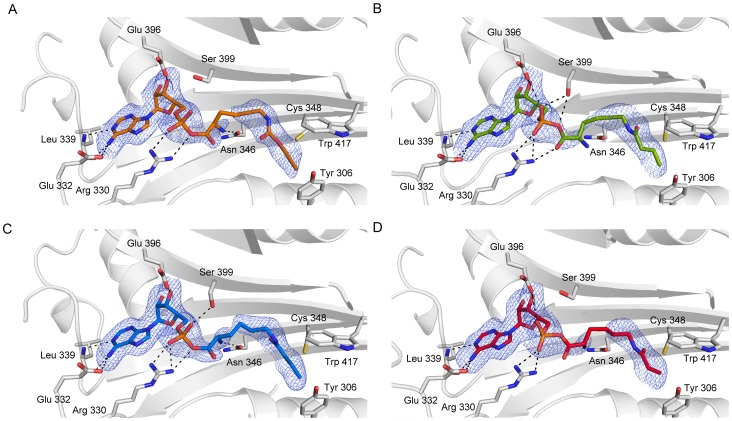
Simulated annealing-omit Fo-*D*Fc electron density contoured at 2 σ of PylRS in complex with (A) Kalk (orange), (B) Kbu (green), (C) Kcr (blue) and (D) Kpr (red). The protein is shown as cartoon, overlaid with its semi-transparent surface representation. Amino acids providing key interactions are drawn as sticks, hydrogen-bonds as dashed lines.

The crystals structures of PylRS in complex with adenylated Kalk, Kbu, Kcr and Kpr (Fig. 1) were solved up to 2.1 Å resolution (Table 1). All four adenylated amino acids occupy the same space in the active site of the aaRS domain (Fig. 2). While the adenosine and phosphate moieties superimpose almost perfectly, the amino group can take up alternative conformations by rotating 180° around the C-Cα bond, as seen in the complex structures of Kalk, Kcr and Kpr. This was previously observed in the structure of the engineered enzyme (PylRS Y306A Y384F) binding *ε-N*-o-azidobenzyloxycarbonyl lysine (PDB code 2ZIO) [30]. In the Wt-Pyl complex the α-amino group of Pyl interacts with the hydroxyl group of Tyr 384 which is located in the β7–β8 hairpin [27]. However, the Pyl analogs lack the ability to form a comparable H-bond and hence this loop is flexible and not defined in the electron density. This flexibility is independent of the bound substrate and it was postulated that this hairpin protects the unstable pyrrolysyladenylate intermediate [25], [26]. In addition, mutation of Tyr 384 to Phe showed that this H-bond interaction between the loop and the substrate is not essential for enzyme function, and its absence may even enhance utilization of non-natural substrates [16], [30]. In the vicinity of the α-phosphate some positive difference density is visible which could correspond to a partly occupied pyrophosphate, reminiscent from the adenylation reaction. Only in the Kpr-complex structure the observed density warranted modeling of the pyrophosphate at 60% occupancy. Compared to the pyrroline ring in Pyl the alkyne, butyryl, crotonyl or propionyl functional groups in Kalk, Kbu Kcr and Kpr are smaller. Thus more space and a higher degree of flexibility is possible and not all atoms of the methylene spacer carrying the functional group are fully defined in the electron density (Fig. 3).

**Table 1:** Data collection, processing and structure refinement statistics.

	Kalk	Kbu	Kcr	Kpr
	(PDB ID 4CH6)	(PDB ID 4CH3)	(PDB ID 4CH4)	(PDB ID 4CH5)
**Wavelength (Å)**	1.0	1.0	1.0	1.0
**Resolution range (Å)**	38.3–2.05(2.12–2.05)	38.4–2.28(2.36–2.28)	42.4–2.16(2.24–2.16)	42.3–2.2(2.28–2.20)
**Space group**	P6_4_	P6_4_	P6_4_	P6_4_
**Unit cell**	104.9 104.9 71.0	105.3 105.3 71.4	105.0 105.0 71.8	105.4 105.4 70.8
**Total reflections**	271,274 (18,606)	123,272 (12,794)	247,942 (23,845)	228,986 (21,051)
**Unique reflections**	28,018 (2,789)	20,634 (2,045)	24,308 (2,439)	22,651 (2,185)
**Multiplicity**	9.7 (6.7)	6.0 (6.3)	10.2 (9.8)	10.1 (9.6)
**Completeness (%)**	100.0 (99.8)	99.9 (100.0)	100.0 (100.0)	99.7 (96.9)
**Mean I/sigma(I)**	45.7 (3.3)	24.7 (3.9)	27.2 (4.5)	23.8 (3.0)
**Wilson B-factor**	43.9	44.3	36.4	43
**R-merge**	0.027 (0.593)	0.044 (0.450)	0.0611 (0.534)	0.0622 (0.699)
**R-meas**	0,029	0,048	0,064	0,066
**CC1/2**	1 (0.877)	1 (0.889)	0.999 (0.923)	0.999 (0.837)
CC*	1 (0.967)	1 (0.97)	1 (0.98)	1 (0.955)
**R-work**	0.174 (0.271)	0.176 (0.231)	0.166 (0.330)	0.174 (0.225)
**R-free**	0.186 (0.296)	0.197 (0.245)	0.209 (0.349)	0.190 (0.257)
**Number of atoms**	2,296	2,252	2,310	2,276
** macromolecules**	2,158	2,151	2,120	2,157
** ligands**	21	21	21	30
** water**	117	80	169	89
**Protein residues**	264	268	259	264
**RMS(bonds)**	0.011	0.01	0.01	0.01
**RMS(angles)**	1.51	1.38	1.4	1.44
**Ramachandran favored (%)**	97	96	97	98
**Ramachandran outliers (%)**	0	0	0	0.4
**Clashscore**	0.23	1.17	0.71	1.62
**Average B-factor**	53.1	49.6	40.1	47.8
** macromolecules**	52.9	49.4	39.3	47.6
** ligands**	58.5	60.3	50.2	58.2
** solvent**	56.0	50.1	49.6	50.1

Numbers in parentheses correspond to the high resolution shell.

### Kalk, Kbu, Kcr and Kpr possess key features required for recognition by PylRS

**Figure 4:**
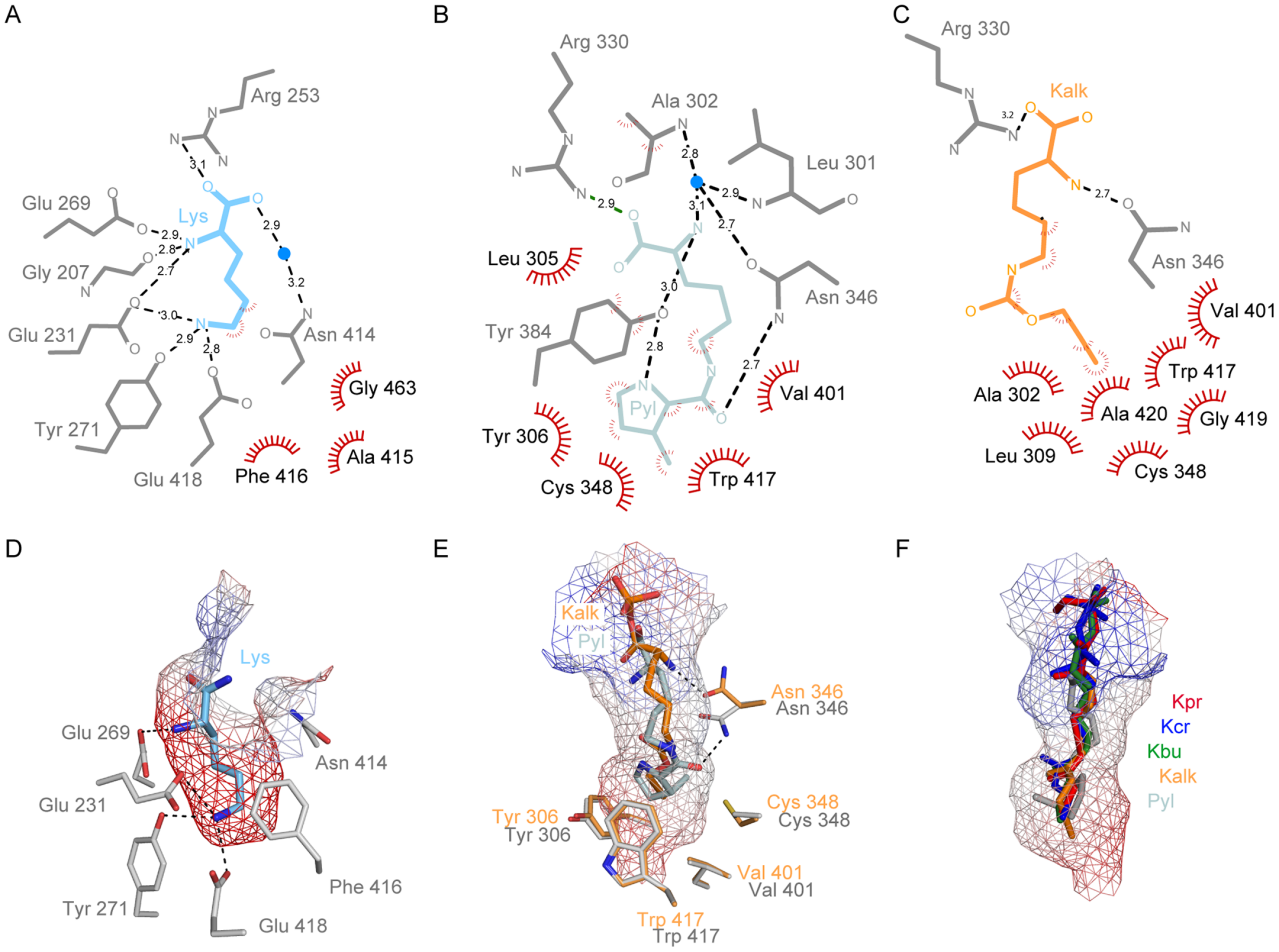
Comparison of the binding pockets of PylRS and LysRS. Two-dimensional plot [52] of residues interacting with Lys (A), Pyl (B) and Kalk (C). Van-der-Waals contacts are shown as red half-spheres. Surface representation of the binding pockets of LysRS (D) and PylRS (E) with Lys, Pyl and Kalk, respectively. (F) Superposition of Pyl (grey), Kalk (orange), Kbu (green), Kcr (blue) and Kpr (red). The surface of the binding pocket is shown as mesh, with the charge distribution indicated by coloring (red  =  negative, blue  =  positive). The surrounding residues are drawn as sticks. (PDB codes PylRS: 2Q7H and LysRS: 3A74).

Both PylRS and lysyl-tRNA-synthetase (LysRS) belong to the type II family of aaRS and thus share the same overall folding topology. A structure of the LysRS from *Bacillus stearothermophilus* (PDB code 3A74), *Bulkholderia thailandensis* (PDB code 4EX5 [38]) and *Escherichia coli* (PDB code 1E22 [39]) are available. The three LysRS share about 53% sequence identity, the catalytic domains superimpose with an r.m.s.d. of 1.2 Å and the residues lining the active site are highly conserved. In comparison, the sequence identity to the catalytic domain of PylRS from *M. mazei* are 17%, 19% and 20%, respectively, and superimpose with an r. m. s. d. of about 2.1 Å. The ATP binding site in both PylRS and LysRS are comparable. The LysRS employs an elaborate interaction network with the Lys and a tight binding pocket (Fig. 4A+D). In contrast, PylRS provides only some key interactions with Pyl in a more spacious, active site lined with hydrophobic residues (Fig. 4B+E). The alkyne group of Kalk, the least flexible of the used derivatives, protrudes slightly deeper into the pocket than Pyl but lies in the same plane as the pyrroline ring. Thus favorable π – π interactions between the amino acid side chain with Tyr 306 as well Trp 417 are possible. (Fig. 4E) PylRS can therefore accommodate a wider range of substrates, if they possess certain key prerequisites: If present, the carbamate, carbonyl or amide moiety, can either interact with Asn 346 or Cys 348, even if this interaction is not permanent, as seen in Pyl [26], [27], [40] and a norbornene containing Pyl analog [28]. For Pyl, an additional hydrogen bond accepting imine nitrogen in the pyrroline ring further increases the activation efficiency [41]. Our data show that analogs containing only the peptide-bond motif, can efficiently bind to the active site of the wild type PylRS. Thus Kalk, Kbu, Kcr, and Kpr are recognized as substrates and fit well in the pocket (Fig. 4F). The preferred substrate head group is hydrophobic and up to four atom bonds in length can be accepted in the binding pocket. Additionally, branched functional groups like a *tert*-butyloxycarbonyl group [30] or small ring systems, such as cyclopentane can be accommodated in the spacious pocket. However, re-engineering of the enzyme active site would be required to accommodate non-natural amino acids lacking these key features. The hot spots which were targeted so far by directed evolution include amino acids whose side chains are either lining the binding pocket (Tyr 306, Leu 309, Asn 346, Cys 348, Trp 417) or resides in the active site closing loop (Ile 417, Phe 384). (Corresponding residues in *M. bakeri*: Leu 274, Asn 311, Cys 313 and Tyr 349) [17], [29], [30], [32]. For instance in order to efficiently use larger and more bulky Pyl-analogues like norbornene or ε-*N-*benzyloxycarbonyl-L-lysine, more room is needed. This can be made available by replacing Tyr 306 at the bottom of the binding pocket by a Gly or Ala [28], [30]. Structural data on PylRS mutants show that even replacing this large amino acid appears not to perturb the overall structural architecture.

### Conclusions

In the last few years numerous PTMs of lysine modifications beyond acetylation and methylation were identified. To understand their function in the epigenetic regulation of biological key processes, the lysine modifying enzymes and their recognition partners need to be identified and their interplay characterized. To address this, an important tool is the site-specific incorporation of these modifications into histones, using orthogonal PylRS:tRNAaaCUA pairs. Here we elucidate the structural basis for the activation of the lysine derivatives Kalk, Kbu, Kcr and Kpr by the wild-type PylRS. This provides further insights into the required features of non-natural amino acids to be utilized by PylRS. Depending on the non-natural amino acid to be incorporated, engineering of PylRS is not necessarily required.

## Material and Methods

### Protein expression, purification and crystallization

Kalk, Kbu, Kcr and Kpr were synthesized as published by Gattner et al and Kaya et al [31], [32]. The sequence encoding the catalytic domain (residues 185–454) of the PylRS from *M. mazei* was PCR amplified and cloned into pET28a, expressed in *E. coli* Rosetta DE3 cells (Novagen) and purified as described previously [27], [28]. In order to co-crystallise PylRS with the Kalk, Kbu, Kcr and Kpr amino acids, the protein was diluted (1 mg mL^−1^) and incubated for 2 h with the respect amino acid (2 mM) and ATP (1 mM; Sigma–Aldrich) in protein storage buffer [HEPES (10 mM, pH 7.4), NaCl (300 mM), MgCl_2_ (5 mM), dithiothreitol (1 mM)]. The protein was concentrated to 10 mg mL^−1^ prior to crystallisation. Crystals appeared overnight in lithium acetate (100 mM) and PEG3350 (10–14 (w/v) %). All crystals were cryoprotected with well solution supplemented with ethylene glycol (30% w/v) before flash-freezing, and then stored in liquid nitrogen until data collection.

### Data collection and structure determination

Diffraction data were collected at the synchrotron beam lines PXI and PXIII (Swiss Light Source, Villigen, Switzerland). The data were processed with XDS [42] to 2.05 Å (Kalk), 2.28 Å (Kbu), 2.15 Å (Kcr) and 2.2 Å (Kpr) spacing, respectively, ensuring consistent indexing and choosing the same set of free reflections. The Crystals belong to the same space group as reported previously for the wild type PylRS [27] (P6_4_, unit cell dimensions: a = b = 105 Å, c = 71 Å). The structure was solved by molecular replacement using the PylRS coordinates (PDB code: 4BW9) in PHASER [43], [44]. For the Kalk, Kcr and Kpr complexes the coordinates of the protein atoms of the PylRS-adenylated Kbu complex were used in rigid body refinement in REFMAC [45]. In order to reduce model bias, all non-protein atoms as well as the loop region around Phe 384 were removed from the model prior to molecular replacement/rigid body refinement, and the temperature factors were reset, followed by simulated annealing in PHENIX [46]. Clear peaks for AMP-Kalk, AMP-Kbu, AMP-Kcr and AMP-Kpr were visible in the simulated-annealing omit Fo-*D*Fc map. Rounds of model building and refinement were carried out in COOT [47] and REFMAC. The refinement parameter file for the adenylated amino acids was generated with prodrg2 [48], as implemented in COOT, and the TLSMD [49] server was used to determine TLS groups for TLS refinement [50]. Diffraction data and refinement statistics are summarized in Table 1. Structural superposition were done with SSM [51]. Two-dimensional interaction plots were carried out with LIGPLOT [52]. All structural figures were prepared with PyMol (Delano Scientific, San Carlos, CA). Atomic coordinates were submitted to the Protein Data Bank (http://www.ebi.ac.uk/pdbe/) with the PDB codes: 4CH6 (PylRS-Kalk), 4CH3 (PylRS-Kbu), 4CH4 (PylRS-Kcr), 4CH5 (PylRS-Kpr)
